# Role of Tula-Family Proteins in Cell Signaling and Activation: Advances and Challenges

**DOI:** 10.3390/ijms25084434

**Published:** 2024-04-18

**Authors:** Alexander Y. Tsygankov

**Affiliations:** Sol Sherry Thrombosis Research Center, Lewis Katz School of Medicine, Temple University, Philadelphia, PA 19140, USA; tsygan@temple.edu

This Special Issue entitled “Role of Tula-Family Proteins in Cell Signaling and Activation: Advances and Challenges” is focused on a relatively novel vertebrate gene/protein family termed alternatively TULA, UBASH3, or STS. This family was described about 20 years ago, and the field of its research saw some major advancements in these two decades. Note that the genes and proteins of this family are annotated in databases as UBASH3A and UBASH3B due to the presence of ubiquitin-associated (UBA) and Src-homology 3 (SH3) domains in their structure, but several synonyms are widely used to denote them. UBASH3A has also been termed STS-2 for suppressor of T-cell signaling, TULA (or TULA-1) for T-cell ubiquitin ligand, and CLIP4 for Cbl-interacting protein 4. UBASH3B has also been termed STS-1, TULA-2, and p70. The terms UBASH3A and UBASH3B will be used throughout this text.

The degree of sequence and structural similarity between UBASH3A and UBASH3B is quite typical for two members of a gene/protein family. In agreement with the structural similarity, these proteins exhibit overlapping functions; thus, both family members possess protein tyrosine phosphatase (PTP) activity, and both suppress protein tyrosine kinase (PTK)-dependent cell signaling [1,2]. However, the members of the UBASH3 family show substantial differences as well; thus, substrate specificity and optimal conditions for their PTP activity differ for UBASH3A and UBASH3B, but even under the respective optimal conditions, UBASH3B is significantly more active than UBASH3A [2,3,4].

Another difference between the two family members is their tissue expression; UBASH3B appears to be more widely expressed than UBASH3A. The former is expressed ubiquitously, while the latter was initially considered lymphoid [5,6,7,8]. However, it has later been shown that UBASH3A is expressed in many myeloid cell types, especially when they are activated [9,10], and even in non-hematopoietic cells [11].

Expression of both UBASH3A and UBASH3B in T lymphocytes and the overlapping effects of these family members on T-cell signaling and responses led researchers to the conclusion that these proteins are important regulators of T-cell functions [4,6,12]. However, if both UBASH3A and UBASH3B regulate T-cell functions by dephosphorylating ZAP-70, a PTK critical for T-cell responses and a well-characterized substrate of these PTPs, one would predict that the effects of both family members on T-cell-dependent biological processes are qualitatively similar and that UBASH3B likely exerts a stronger effect due to its higher PTP activity. Consistent with this notion, studies in platelets demonstrated that UBASH3B alone can strongly regulate signal transduction mediated by the Syk PTK, which belongs to the same family as ZAP-70 and is very similar to the latter [13].

Despite these hypothetic expectations, it has been shown that UBASH3A is involved in many autoimmune diseases, including such serious T-cell-dependent conditions as type 1 diabetes [14,15,16,17,18], rheumatoid arthritis [19,20,21], and systemic lupus erythematosus [22,23,24], while the only such link for UBASH3B appears to be restricted to Behcet’s disease, a chronic inflammatory condition [25,26]. Consistent with the data on autoimmunity, the comparison of knockout mice lacking UBASH3A and/or UBASH3B in colitis models indicated that the effects of single knockouts on experimental inflammation are qualitatively different [12]. Together, these results suggest that family members can also influence T-cell responses through mechanisms different from their PTP activity. Indeed, UBASH3B has been shown to possess phosphoesterase activity that exerts a negative effect on T-cell signaling and activation [27], although this finding cannot explain the unique link of UBASH3A to T-cell-dependent autoimmune conditions.

A possible explanation of the specificity of UBASH3A/B links to autoimmunity may lie in the findings that UBASH3A influences T-cell functions not only through the effects overlapping with those of UBASH3B but also through UBASH3A-specific ones. Thus, UBASH3A has been shown to interfere with NF-κB signaling by directly interacting with and inhibiting the I-κB kinase complex [15] and to down-regulate the surface level of the T-cell antigen receptor [28]. These findings may explain why UBASH3A is more important in autoimmunity. It remains to be understood what mechanisms mediate the specific involvement of UBASH3B in Behcet’s disease [25,26], which contrasts with the absence of a link between (i) UBASH3B and most known autoimmune conditions and (ii) UBASH3A and Behcet’s disease.

It should also be noted that despite the important contribution of both family members in biological processes mediated by hematopoietic cells, such as immunity (see above), hemostasis, and thrombosis [13,29], as well as hematopoietic cancers [30,31], they appear involved in various functions and pathologies of non-hematopoietic cells ranging from promoting invasiveness and metastasis of breast cancer [32] to regulation of individual development [11].

All these findings greatly expand the initial view of a lymphoid-specific and ubiquitously expressed family member exerting overall similar effects in different cellular systems; it appears that despite notable similarities, the two proteins possess unique features, allowing them to produce specific effects that are not limited to a particular cell type. It has been suggested that fundamental issues related to the specificity of UBASH3A/B functions may be addressed through the comparative genetic and biochemical analysis of this family.

This analysis indicates that the presence of a UBASH3-like gene is typical for all invertebrates, while the two-member UBASH3 family is typical for vertebrate species. This family is highly conserved in such diverse taxa as mammals and other tetrapods, lobe-finned fish, which are considered ‘living fossils’, and sharks [33]. These findings imply that the systems specific to vertebrates, including adaptive immunity with its lymphocytes and the closed circulatory system with its thrombocytes and platelets, require this two-member family instead of a single protein for building the respective regulatory circuits.

However, this comparative analysis identifies atypical taxa as well; UBASH3A appears to be lost at the root of the ray-finned fish tree; thus, some ray-finned fish species thrive possessing only a single family member. Further analysis suggests that the loss of UBASH3A is followed by the formation of a unique UBASH3 family in teleosts, which possess two or even three UBASH3B-related loci [33]. This atypical UBASH3 family differs dramatically from its conserved form found in most vertebrates, although the immune system of teleosts is similar to that of other vertebrates, including mammals [34,35,36], in which both UBASH3A and UBASH3B play important and, to a substantial extent, specific roles. Whether these findings suggest that the specific functions of UBASH3A are not essential for cellular regulation or that the immune responses in teleost fish significantly differ from those in other vertebrates remains to be determined.

The papers published in this Special Issue aimed to summarize the accumulated knowledge and present several key issues of the current research in this area. The review by Zaman, French, and Carpino is focused on the role of UBASH3A/B proteins in immunity, undoubtedly their best-characterized function [37]. The effects of UBASH3A and UBASH3B on T-cell signaling and responses, which have been shown in the seminal studies [1,6], are discussed to characterize the mechanistic link between these functions and the biochemical and structural properties of the family members. Of particular interest are the studies of the effects of UBASH3A/B proteins on innate immune responses to infections; they appear to be specific for different cell types, and the contributions of individual family members to these effects appear to be distinct [37].

As mentioned above, UBASH3A uniquely contributes to the development of autoimmune conditions, such as type 1 diabetes (T1D), by exerting effects on T-cell responses [15,28]. The study by Newman, Concannon, and Ge [38] demonstrates the interaction of UBASH3A and PTPN22 (also known as PEP or Lyp) on the following several levels: (i) these PTPs physically associate via the UBASH3A SH3 domain; (ii) UBASH3A and PTPN22 transcripts cooperatively regulate IL-2 expression by T-cells from T1D cases; and (iii) genetic risk variants in *ubash*3a and *ptpn*22 statistically interact, jointly affecting the risk of T1D.

The effects of UBASH3A/B on cell signaling and responses are also reviewed by Tsygankov [33]. In addition to the effects of both family members on immune cells, the effects of UBASH3B on platelets, which appear to play an important role in the regulation of hemostasis and thrombosis [13,29], are discussed. In particular, the role of Syk-family PTKs in mediating the biological effects of UBAH3A/B and the dephosphorylation of individual regulatory sites of Syk as the molecular basis of this role are discussed in this review. Finally, the comparative biochemistry approach is presented as a tool for further understanding the biological functions of the UBASH3 family [33].

The review by Vukojevic et al. [39] expands the discussion of the role of UBASH3 proteins into the realm of individual development. Although this role of the UBASH3 family remains insufficiently understood, some recent data [11] strongly suggest that UBASH3A acts as a regulator of renal development and a viability factor in renal cells, possibly through its interactions with Syk [39], a rather ubiquitous PTK that plays a key role in many biological processes.

This and other cellular roles of UBASH3 proteins may be mediated not only by the regulatory dephosphorylation of Syk but also by the adaptor functions based on the ability of UBASH3A/B to form complexes with a wide variety of proteins [3,7,29,40,41,42,43]. The review by Hayes and van der Geer [44] focuses on the current state of knowledge in this area. Physical and functional interactions between UBASH3A/B and various proteins, including kinases, adaptors, and trafficking factors, are analyzed, and the role of these interactions in the biological functions of UBASH3A/B is discussed.

To sum up, this Special Issue presents the steady advance in understanding the TULA/UBASH3/STS family from a mysterious, if not orphaned, object to a well-characterized section of life sciences. However, the papers published here also reveal considerable challenges in understanding this family, which, despite being thoroughly studied for two decades, resists simplification and remains rather enigmatic.
